# Supplementary material for: The Heteromeric Dopamine Receptor D2:D3 Controls the Gut Recruitment and Suppressive Activity of Regulatory T-Cells
Source: Int J Mol Sci. 2025 Oct 16;26(20):10069. doi: 10.3390/ijms262010069 (PMC12563322; doi:10.3390/ijms262010069)
Supplement: Supplementary file 1 [file ijms-26-10069-s001.zip › ijms-3840451-supplementary.pdf]

Supplementary figures

The heteromeric dopamine receptor D2:D3 controls the gut recruitment and suppressive activity of regulatory T-cells

Jacob Mora, Iu Raïch, Valentina Ugalde, Gemma Navarro, Carolina Prado, Pia M. Vidal, Pedro Leal, Alexandra Espinoza, Moting Liu, Rinse Weersma, Ranko Gacesa, Marcela A. Hermoso, Rafael Franco, Rodrigo Pacheco

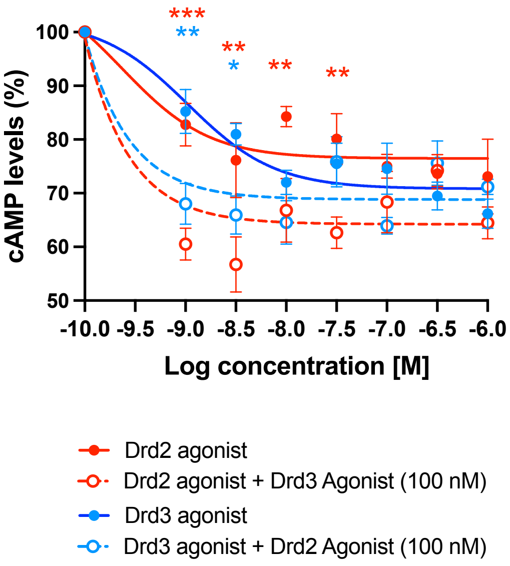

Figure S1. Drd2 and Drd3 stimulation synergise in the inhibition of cAMP production. HEK293T cells were transfected with Drd2 and Drd3 and incubated for 48h. Afterward, cells were pre-incubated in serum-free medium for 4h, seeded in white ProxiPlate 384-well microplates (10^3 cells/well) and then treated with indicated concentrations of 7-OH-PIPAT maleate (Drd3-agonist) or sumanirole (Drd2-agonists) each alone or together for 15 mins. Cells were then treated with 500 nM forskolin for 15 mins. cAMP production was quantified by a TR-FRET methodology. Data is represented as % of cAMP accumulation. Values are mean ± SEM of triplicate from a representative of three independent experiments. Mean ± SEM are indicated. *, p<0.05; **, p<0.01; ***, p<0.001 by two-way ANOVA followed by Tukey's post-hoc test. Red asterisks indicate differences between red groups, and blue asterisks indicate differences between blue groups.

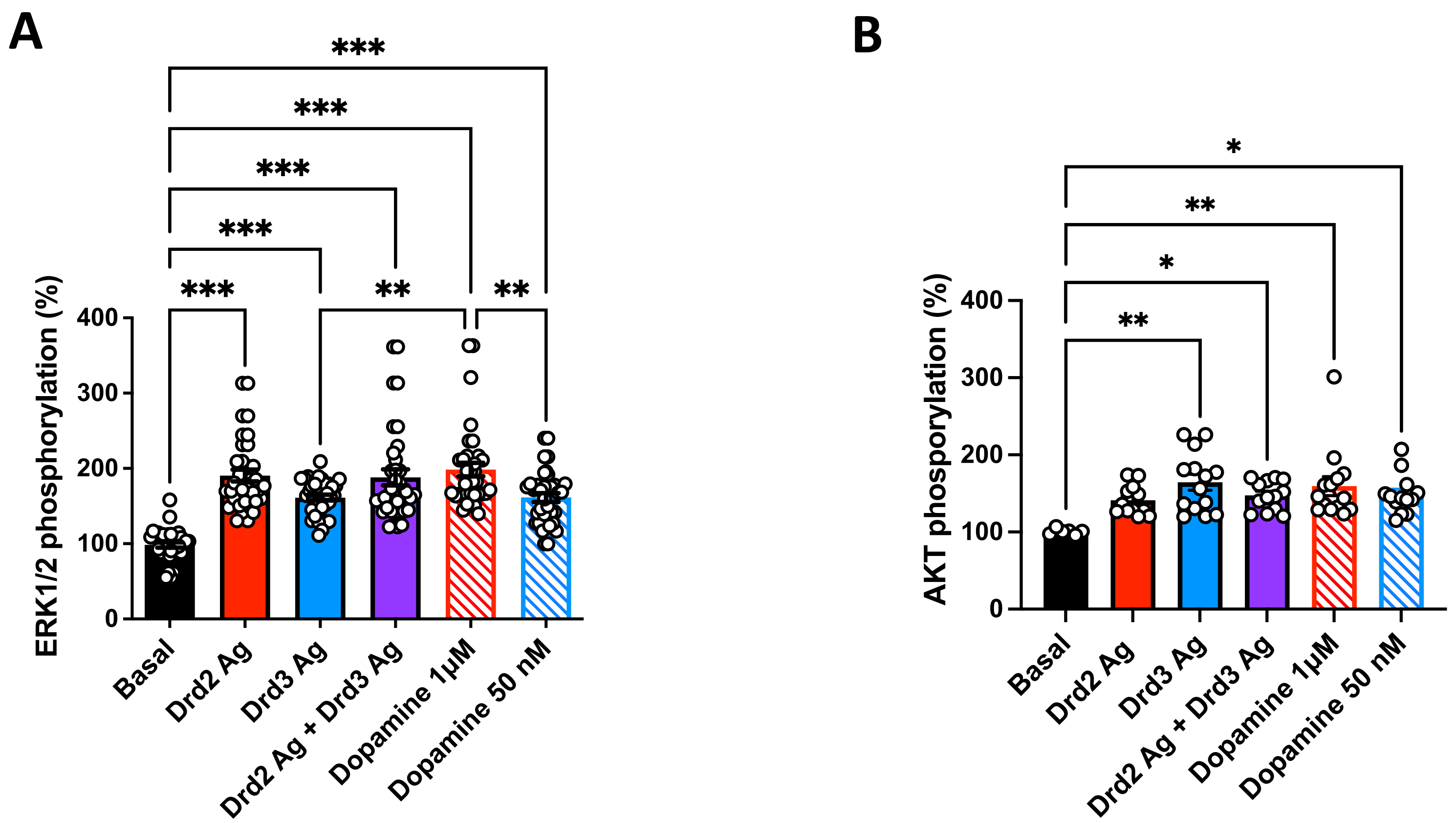

Figure S2. Analysing the cross-talk of Drd2 and Drd3 at the level of signalling pathways. HEK293T cells were transfected with Drd2 and Drd3 and incubated for 48h. Afterward, cells were pre-incubated in serum-free medium for 4h, seeded in white ProxiPlate 384-well microplates (10^3 cells/well) and then treated with dopamine (50 nM or 1000 nM), 7-OH-PIPAT maleate (Drd3-agonist; 100 nM) or sumanirole (Drd2-agonists; 100 nM) each alone or together for 15 mins. Phosphorylation of ERK1/2 (**A**) or AKT (**B**) were determined by alpha-screen bead-based technology. Each symbol represents data obtained from an individual determination (n = 36 in A; n = 9-15 in B). Mean ± SEM are indicated. *, *p*<0.05; **, *p*<0.01; ***, *p*<0.001 by one-way ANOVA followed by Bonferroni's post-hoc test.

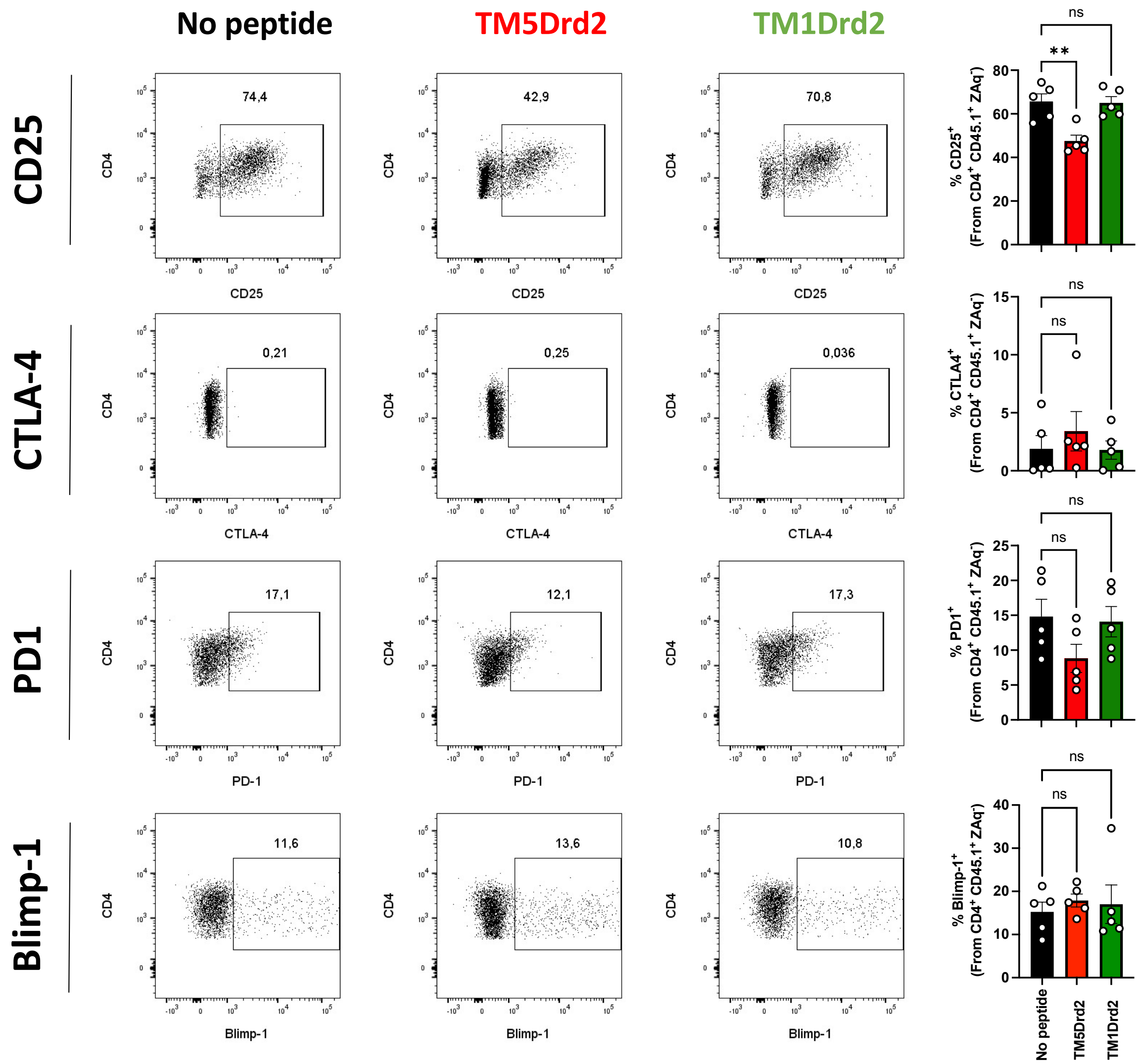

Figure S3. Disassembling of the Drd2:Drd3 heteromer selectively reduces CD25 expression on Treg. Splenic Treg cells ($CD4^{+}GFP^{+}$) isolated from *CD45.1*$^{+/+}$ *Foxp3*^{gfp} mice were incubated with 4μM TM1Drd2 (green), TM5Drd2 (red) peptides or only vehicle (black) for 4h. During the last 30 min, cells were treated with dopamine 2 μM. Naïve $CD4^{+}$ $CD25^{-}$ T-cells (T naive) isolated from WT *CD45.2*$^{+/+}$ mice were activated with anti-CD3 and anti-CD28 Abs in the presence of peptide-treated Treg at a Tnaive:Treg ratio of 2:1. After 72h, the expression of CD25, CTLA-4, PD1, and Blpim-1 was determined in the $CD4^{+}$ $CD45.1^{+}$ ZAq^{-} population by flow cytometry. Left panels show representative dot plots. Numbers on the plots indicate the percentage of cells inside the region. Right panels show the quantification determined as the % of cells expressing the corresponding marker. Values are the mean ± SEM from five independent determinations. Each symbol represent the data from an independent individual. **, p<0.01 by one-way ANOVA followed by Tukey's post-hoc test. ns, non-significant.

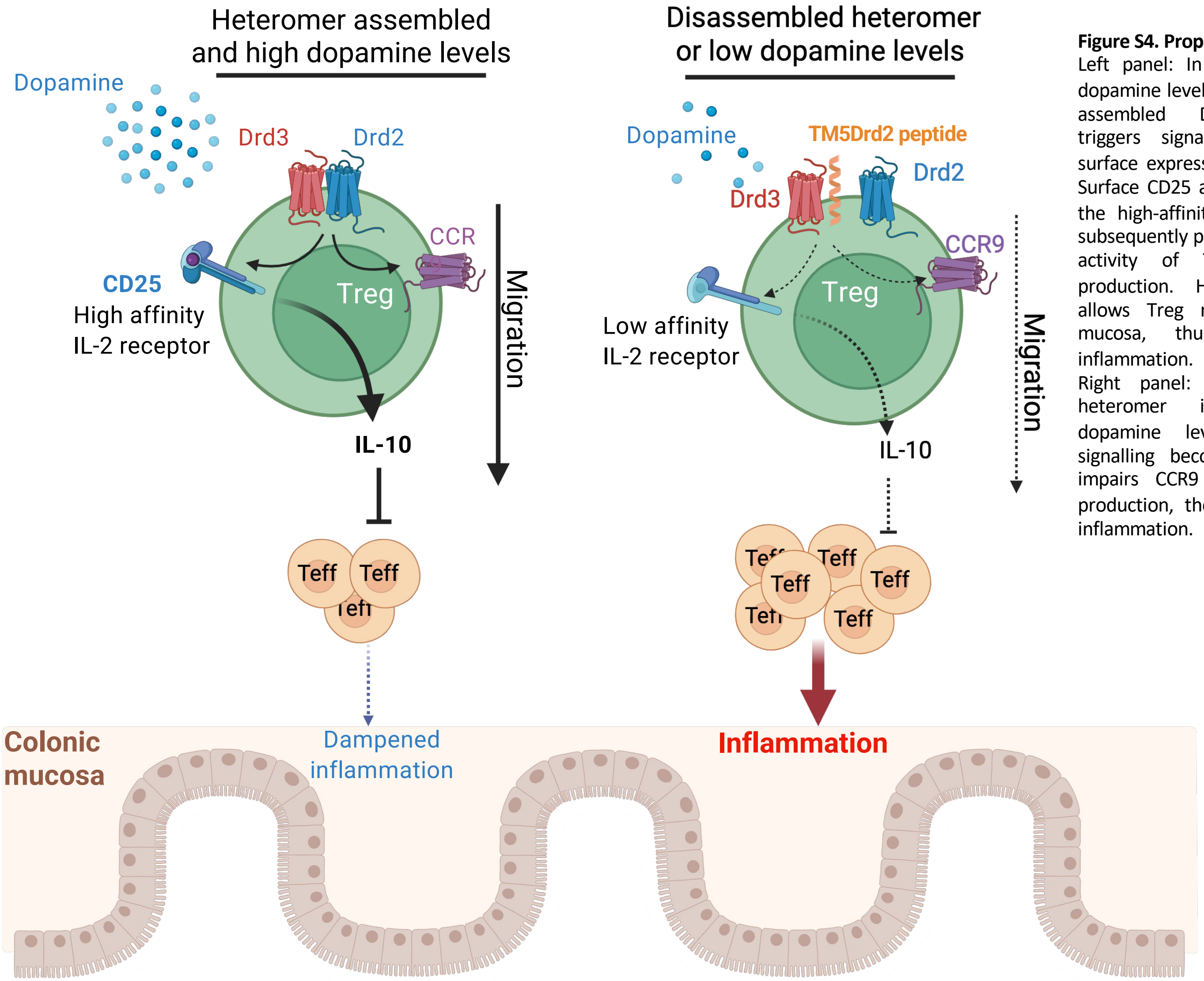

Figure S4. Proposed model.
Left panel: In the presence of high dopamine levels, the stimulation of the assembled Drd2:Drd3 heteromer triggers signalling to induce high surface expression of CD25 and CCR9. Surface CD25 allows the expression of the high-affinity IL-2 receptor, which subsequently promotes the suppressive activity of Treg, including IL-10 production. High CCR9 expression allows Treg migration towards gut mucosa, thus limiting intestinal inflammation.
Right panel: when the Drd2:Drd3 heteromer is disassembled or dopamine levels are low, Drd3 signalling becomes dominant, which impairs CCR9 expression and IL-10 production, thereby favouring colonic inflammation.
